# MKS proteins regulate centrosome positioning during cell migration

**DOI:** 10.1186/2046-2530-1-S1-P65

**Published:** 2012-11-16

**Authors:** H Dawe, D Pitcher, AR Barker, M Moles

**Affiliations:** 1 University of Exeter, UK

## 

Meckel-Gruber syndrome (MKS) is a severe ciliopathy, but how the underlying mutations lead to the disease is poorly understood. MKS proteins form complexes at the ciliary transition zone, and are also needed for centrosome re-orientation and docking with the plasma membrane during ciliogenesis. However, MKS-mutant cells also have an unexplained cell migration defect: cells can migrate, but the rate is much slower than usually observed. As correct centrosome orientation is vital for many migrating cells, we examined centrosome appearance and position in migrating patient fibroblasts with mutations in *MKS2* and *MKS3*. In control cells, the centrosome and Golgi apparatus were located between the nucleus and the leading edge, but these organelles were randomly positioned in patient fibroblasts. To distinguish between failure and delay in centrosome polarisation, we assessed centrosome position during the first two hours of cell migration. We found that mutant fibroblasts failed to stably re-orient the centrosome over the time-course of the assay, suggesting this may be a contributing factor to the migration defect. Finally, we asked if a cilium is needed for correct centrosome polarisation. Wild-type fibroblasts re-oriented their centrosome in the absence of a cilium, indicating that direction sensing by the primary cilium is not needed for initial centrosome polarisation in these cells. As MKS cells are defective in centrosome positioning during migration as well as ciliogenesis, we conclude that the MKS ciliogenesis defect may not be the primary cause of disease, but a downstream consequence of the failure to correctly position the centrosome.

